# Supplementary material for: Observation of an exotic state of water in the hydrophilic nanospace of porous coordination polymers
Source: Commun Chem. 2020 Feb 7;3:16. doi: 10.1038/s42004-020-0262-9 (PMC9814769; doi:10.1038/s42004-020-0262-9)
Supplement: Supplementary file 1 — Description of Additional Supplementary Files [file 42004_2020_262_MOESM1_ESM.pdf]

Description of Additional Supplementary Files

File Name: Supplementary Data 1
Description: Crystallographic information file (CIF) of PCP-1 (CCDC number:1893959)
