# Supplementary material for: Observation of an exotic state of water in the hydrophilic nanospace of porous coordination polymers
Source: Commun Chem. 2020 Feb 7;3:16. doi: 10.1038/s42004-020-0262-9 (PMC9814769; doi:10.1038/s42004-020-0262-9)
Supplement: Supplementary file 2 — Supplementary Information [file 42004_2020_262_MOESM2_ESM.pdf]

Supplementary Information

Observation of an exotic state of water in the hydrophilic nanospace of porous coordination polymers

Tomoaki Ichii[1*], Takashi Arikawa[1], Kenichiro Omoto[2], Nobuhiko Hosono[2], Hiroshi Sato[2], Susumu Kitagawa[2], Koichiro Tanaka,[1,2*]

*Correspondence to: kochan@scphys.kyoto-u.ac.jp (K.T.); ichii.tomoaki.46v@st.kyoto-u.ac.jp (T.I.)

Supplementary Methods

Materials

All solvents, organic and inorganic reagents are commercially available, and were used without further purification. Single crystals of **PCP-1** were prepared according to previously reported procedures.[1] Microwave syntheses were performed using Biotage model Initiator+. Single-crystal X-ray crystallographic analysis was performed using Rigaku model XtaLab P200 diffractometer equipped with a Dectoris PILATUS 200 K detector, using a VariMax Mo Optic with MoKα radiation (λ = 0.71075 Å) and a confocal monochromator. The obtained data were calculated using a CrystalStructure (Rigaku) crystallographic software package, SHELXL-97[2], except for refinement. All non-hydrogen atoms except O-atoms of guest H_2O were refined using anisotropic thermal parameters. Powder X-ray diffraction data were collected with a Rigaku model SmartLab X-ray diffractometer with CuKα radiation (λ = 1.541 Å) equipped with a temperature controller under dry N_2 atmosphere unless mentioned. Thermogravimetric analyses (TGA) were performed on a Rigaku model Thermo plus EVO under dry N_2 atmosphere. The H_2O and D_2O sorption isotherms were obtained using a MicrotracBEL model BELSORP-aqua3 instrument.

Synthesis of single crystals of Cu(ipa)·$3H_2O$ (**PCP-1**)

5-Methylisopthalic acid (H_2ipa) (272 mg, 1.51 mmol, 1.0 eq) and H_2O (10 mL) were placed in a pressure vessel, which was stirred at 150 ºC for 1 h using a microwave reactor. After cooling down to room temperature, to the mixture was added $Cu(NO_3)_2\cdot 2.5H_2O$ (353 mg, 1.51 mmol, 1.0 eq) in H_2O (10 mL). After standing the mixture at 150 ºC for 5 days, Cu(ipa)·$3H_2O$ (**PCP-1**) was obtained as blue block single crystals. After washing with H_2O, the resultant single crystals were stored in H_2O. Crystal structure of **PCP-1** is described on Supplementary Figure S1.

Result of single-crystal X-ray crystallographic analysis for **PCP-1** at 298 K

Crystal data for $C_9H_{12}CuO_7$: F_w = 295.74, tetragonal, space group P4/nmm, a = b = 19.0629(3) , c = 6.91407(17) Å, V = 2512.53(8) $Å^3$, Z = 8, ρ_{calcd} = 1.563 gcm^{-3}, μ = 1.7571 mm^{-1}, T = 298 K, λ(MoKα) = 0.71075 Å, $2\theta_{max}$ = 50.0°, 17310/1232reflection collected/unique (R_{int} = 0.0221), R_1 = 0.0384 ($I > 2\sigma(I)$), wR_2 = 0.1243 (for all data), GOF = 1.079, largest diff. peak and hole 0.82 / –0.75 $eÅ^{-3}$. Hydrogen atoms of H_2O molecules were not assigned. CCDC deposit number 1893959.

PXRD of powder crystals of **PCP-1** and **PCP-2**

Reversible H_2O desorption/adsorption behaviors of **PCP-1** and **PCP-2** were monitored by PXRD under open air or dry N_2 atmosphere at 30 ºC (Supplementary Figure S4). For H_2O desorption, powder crystals of **PCP-1** were exposed to dry N_2 atmosphere for ca. 2 h at 30 ºC to remove guest H_2O to provide powder crystals of **PCP-2**. PXRD of the resultant **PCP-2** were measured under dry N_2 atmosphere at 30 ºC (Supplementary Figure S4c). For H_2O adsorption, resultant powder crystals of **PCP-2** were exposed to open air for ca. 0.5 h at 30 ºC to adsorb guest H_2O to provide **PCP-1**. PXRD of the resultant powder crystal were measured under open air at 30 ºC (Supplementary Figure S4d).

Upon exposure of **PCP-1** to dry N_2 atmosphere at 30 ºC for ca. 2 h, PXRD pattern of **PCP-1** changed to that of **PCP-2**, as evidenced by a distinct increase in the intensity of 110 diffraction (2θ = 6.5º, Supplementary Figure S4b,c). Furthermore, upon exposure of the resultant powder crystals of **PCP-2** to open air, PXRD pattern of **PCP-1** restored, as evidenced by the decrease in 110 diffraction (2θ = 6.5º, Supplementary Figure S4c,d). These reversible changes in the PXRD patterns suggest that **PCP-1** reversibly releases/captures the guest H_2O upon exposure to dry N_2 atmosphere or ambient air at 30 ºC without degradation of its host framework.

SEM observation of **PCP-1.**

Field-Emission Scanning electron microscopy (FE-SEM) observations of powdery PCP-1 were performed with a HITACHI model SU-5000 operating at 15 kV. The dried samples deposited on carbon tape were coated with osmium using an osmium coater (Vacuum Device model HPC-20) prior to the observations.

Synthesis of powder crystals of Cu(ipa)•3H_2O (**PCP-1**) and Cu(ipa)•H_2O (**PCP-2**)

5-Methylisopthalic acid (H_2ipa) (195 mg, 1.1 mmol, 1.0 eq) and H_2O (54 mL) were placed in a pressure vessel, which were stirred at 150 ºC for 1 h using a microwave reactor. After cooling down to room temperature, to the mixture was added $Cu(NO_3)_2$•2.5H_2O (256 mg, 1.1 mmol, 1.0 eq) and pyridine (80 µL) in H_2O (10 mL). After stirring of the mixture at 150 ºC for 1.5 h, blue powder crystals were obtained. The resultant powder crystals were collected by decantation, washed successively with EtOH (7.5 mL x 3) and H_2O (15 mL x 3), and dried under ambient air to afford Cu(ipa)•3H_2O (**PCP-1**) (77.67 mg, 0.26 mmol, 24%) as blue powder crystals. The PXRD and TGA of the resultant material supported formation of **PCP-1** in powdery form (Supplementary Figure S3, S6).

The guest H_2O of the powder crystals of **PCP-1** were selectively removed upon standing **PCP-1** under dry N_2 atmosphere or reduced pressure around room temperature, allowing us to obtain the monohydrated product, Cu(ipa)•H_2O (**PCP-2**), as characterized by following PXRD and TGA data (Supplementary Figure S3, S6).

TGA of powder crystals of **PCP-1**

TG profiles of powder crystals of **PCP-1** were obtained in two distinct methods; measured with (i) constant and (ii) valuable heating-rate experiments.

(i) TGA of powder crystals of **PCP-1** measured with constant heating rate (5 ºC/min)

TG profile of **PCP-1** (Supplementary Figure S2) measured with constant heating rate (5 ºC/min) showed two distinct weight losses around 30–70 °C (**A**) and 70–120 °C (**B**), corresponding to the losses of the guest H_2O (2 molecules per one Cu(II)) and the coordinating H_2O (1 molecule per one Cu(II)), respectively(*32*). The decomposition of the sample beyond 300 ºC is confirmed by the sharp weight loss in the TG profile.

(ii) TGA of powder crystals of **PCP-1** measured with valuable heating rate

Powder crystals of **PCP-1** were firstly exposed to dry N_2 atmosphere with a constant temperature condition (30 ºC) for 240 min, then the temperature was increased up to 500 ºC with the heating rate of 5 ºC/min. The weight reduction of the sample was monitored in time course as described on Supplementary Figure S3.

· **0–240 min (constant temperature condition (30 ºC))** Upon exposure of the sample to dry N_2 atmosphere at 30 ºC, a definite weight loss (**C**: 2 H_2O per one Cu(II) center) followed by plateau was observed.

· **240 min–330 min (heating at 5 ºC/min)** Upon increasing the temperature from 30 ºC with the heating rate of 5 ºC/min, a definite weight loss (**D**: 1 H_2O per one Cu(II) enter) followed by plateau was observed.

The weight loss followed by plateau observed as **C** (2 H_2O per one Cu(II) center) suggests **PCP-1** selectively released guest H_2O upon exposure to dry N_2 atmosphere at 30 ºC to afford **PCP-2**. The weight loss observed as **D** (1 H_2O per one Cu(II) enter) suggests loss of coordinating H_2O from **PCP-2** upon heating. The decomposition of the sample beyond 300 ºC (300 min) is confirmed by the sharp weight loss in the TG profile.

Isotope effects on peak widths of coordinated water in **PCP-2**

The peaks H1 and H2 can be assigned to the stretching modes of H-bonded OH (HB-OH) and non-H-bonded OH (Free-OH), respectively. This is also confirmed by the isotope effect on their peak widths. The widths of the absorption peaks due to the HB-OH stretching modes are strongly affected by the anharmonic coupling with lower frequency modes such as O-O vibration modes[3]. The full width at half maximum (FWHM) is proportional to the reciprocal of the mass of hydrogen atom[4,5]. This suggests that the FWHM of HB-OH should be reduced by a factor of 2 by the isotope substitution. To confirm this point, we performed spectral shape analysis of the absorption peaks. Each peak is nicely described by the Lorentz function as shown by the black curves in Fig. 3a, which means the structural inhomogeneity of the coordinated water is negligible. This reflects the high crystallinity of the **PCP-2** framework. The fitting results are summarized in Table 1. The ratio of FWHMs between H1 and D1 is 140/73.5 = 1.90, which strongly suggests that peak H1 (D1) is due to HB-OH (HB-OD). In contrast, the FWHMs of H2 and D2 are almost the same (33.5/36.8 = 0.91), indicating that H2 (D2) is due to Free-OH (Free-OD).

Number of Free-OH and HB-OH arms in **PCP-2**

The absorbance area, when normalized by the oscillator strength, tells us the number of Free-OH and HB-OH arms in the **PCP-2**. Table 1 shows the absorbance area of HB-OH is 4.8 time larger than that of Free-OH. This is almost perfectly compensated by the increase in the oscillator strength by a factor of 5 ± 0.4 due to the H-bonding[6]. After all, the IR absorption data suggests that there are equal number of HB-OH and Free-OH arms. Considering the structural homogeneity, this result indicates that every single coordinated water molecule has both HB-OH and Free-OH arms.

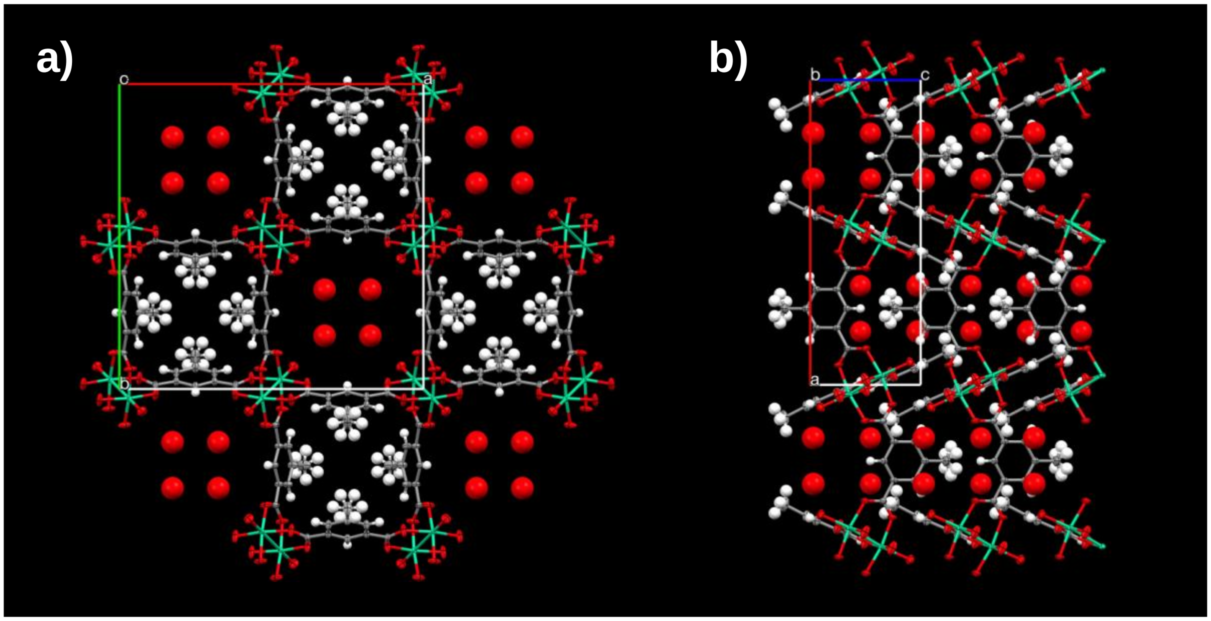

Supplementary Figure 1.
ORTEP views (50% probability level) of **PCP-1** based on single-crystal X-ray crystallographic analysis. Views from a) *c*-axis and b) *b*-axis. (C: gray, Cu: pale green, O: red, H: white).

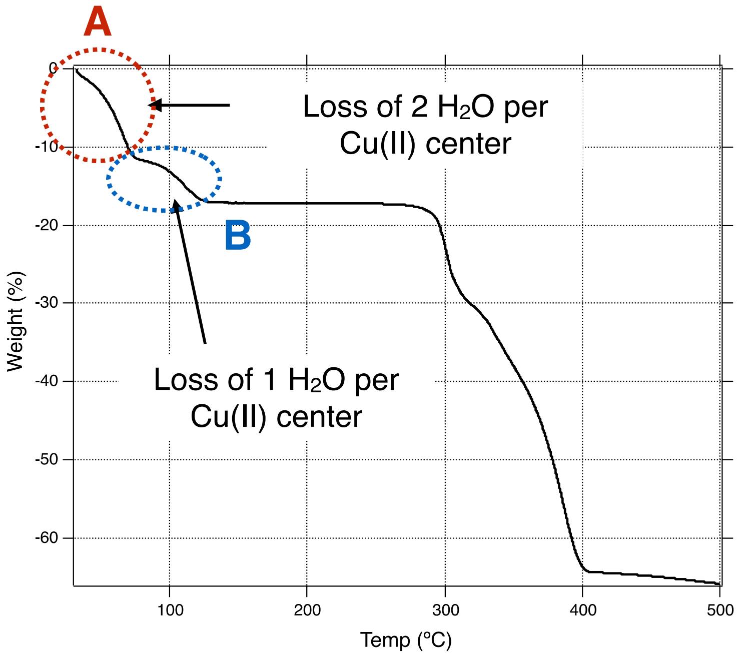

Supplementary Figure 2.
TG profile of the powder crystals of **PCP-1** measured with constant heating rate of 5 ºC/min under N_2 atmosphere.

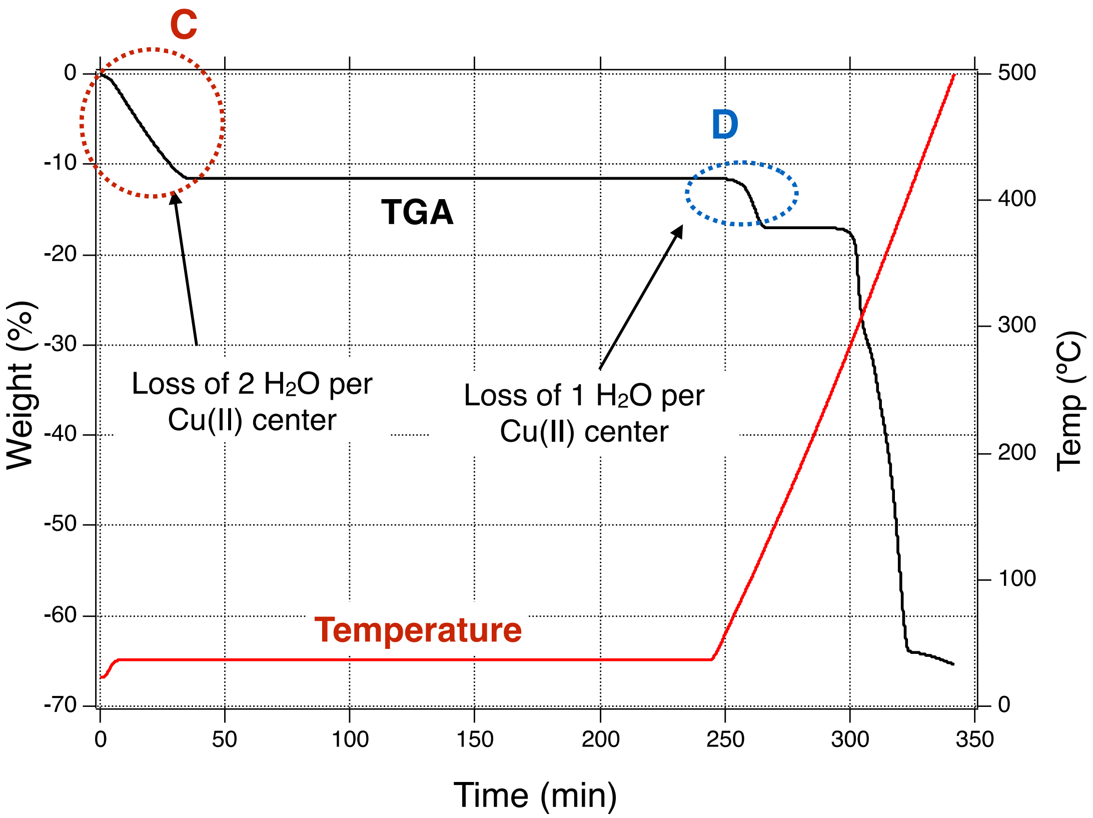

Supplementary Figure 3.
TGA profile of the powder crystals of **PCP-1** measured with variable heating conditions under N_2 atmosphere (**0 ~ 240 min**: 30 ºC (constant), **240 min ~ 330 min**: heating late 5 ºC/min.).

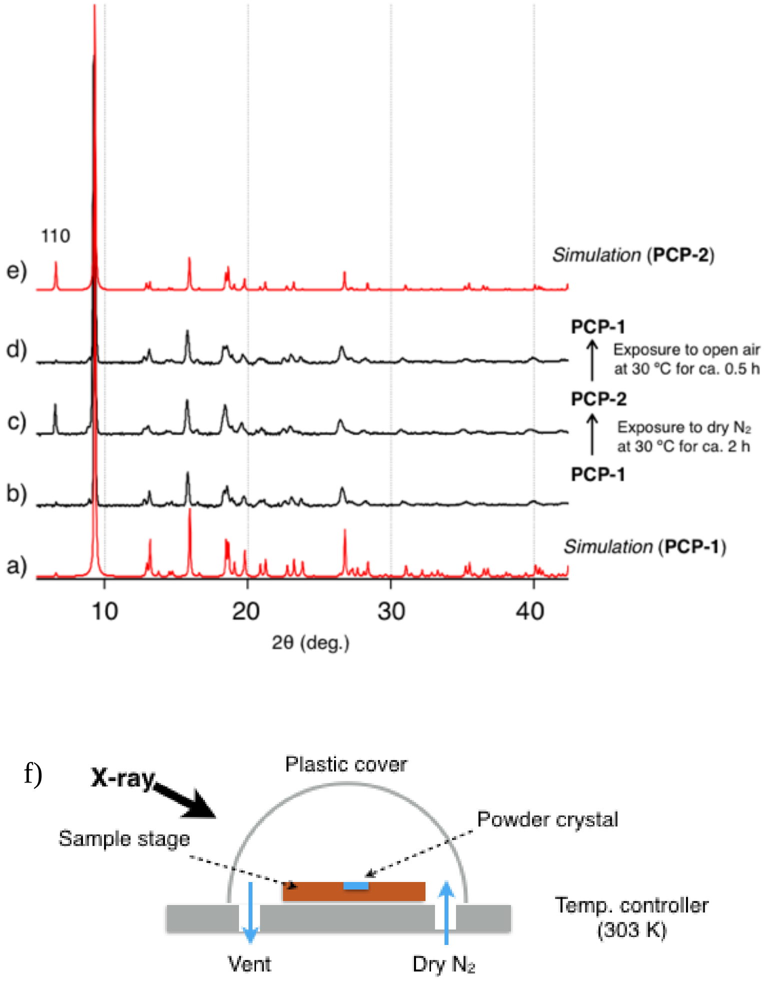

Supplementary Figure 4.
PXRD profiles of **PCP-1** and **PCP-2** (CuKα, λ = 1.541 Å, 30 ºC). Simulated powder XRD patterns of a) **PCP-1** and e) **PCP-2**. Experimental powder XRD patterns of b) **PCP-1**, c) **PCP-2** prepared by exposing powder crystals of **PCP-1** to dry N_2 atmosphere at 30 ºC for ca. 2 h, and d) **PCP-1** prepared by exposing powder crystals of **PCP-2** to open air at 30 ºC for ca. 0.5 h. (f**)** Schematic illustration of a sample stage for PXRD measurements equipped with a temperature controller, dry N_2 inlet, and a plastic cover.

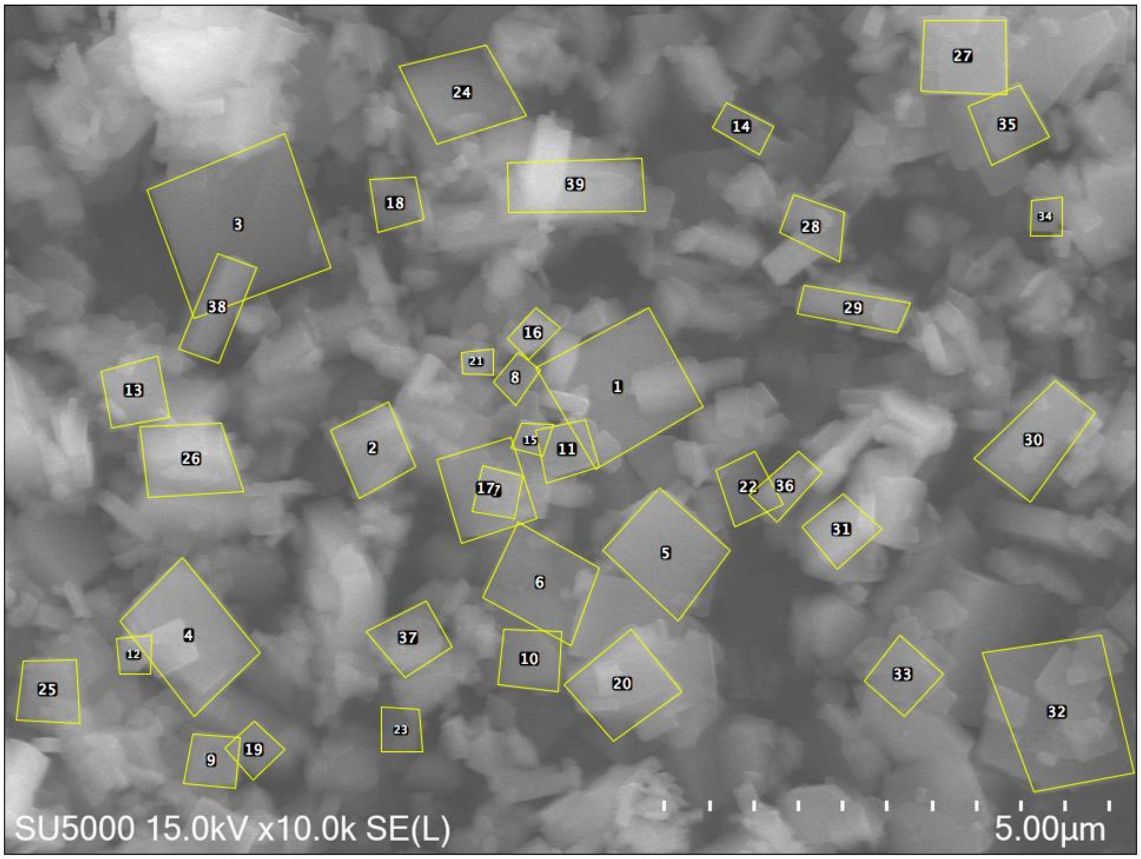

Supplementary Figure 5.
Image of **PCP-1** crystals taken by Scanning Electron Microscope (SEM). The average value of area is 0.64 µm2.

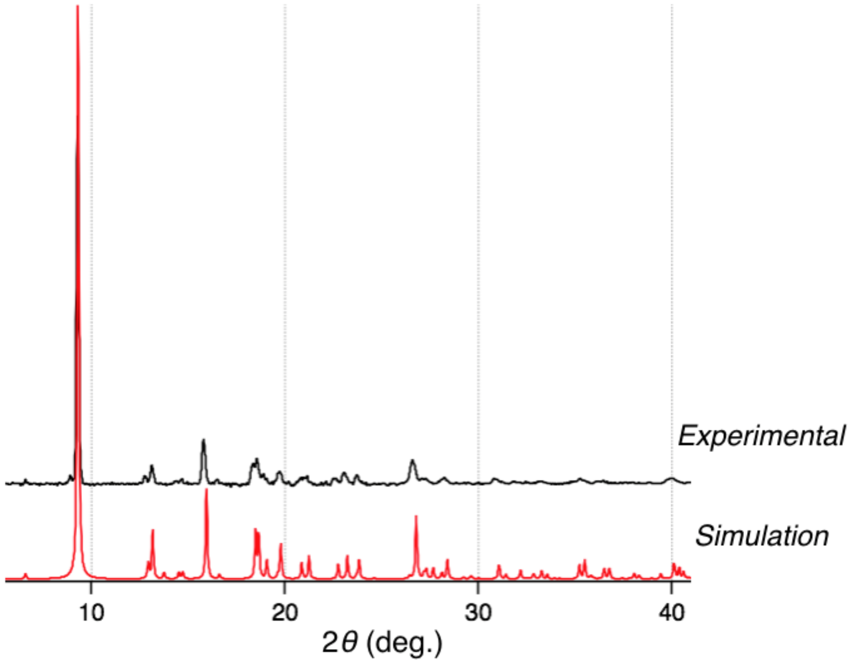

Supplementary Figure 6.
PXRD profile of the powder crystals of **PCP-1** (30 ºC, CuKα, λ = 1.541 Å).

Supplementary References

1. Zou, R. Q., Sakurai, H., Han, S., Zhong, R. Q. & Xu, Q. Probing the Lewis acid sites and CO catalytic oxidation activity of the porous metal-organic polymer [Cu(5-methylisophthalate)]. *J. Am. Chem. Soc.* **129**, 8402–8403 (2007).
2. Sheldrick, G. M. *Program for refinement of crystal structures*. (University of Gottingen, Germany, 2013).
3. Bonn, M., Brugmans, M. J. P., Kleyn, A. W., Van Santen, R. A. & Bakker, H. J. Vibrational dephasing mechanisms in hydrogen-bonded systems. *Phys. Rev. Lett.* **76**, 2440–2443 (1996).
4. Yarwood, J. & Robertson, G. N. A new method of measuring the hydrogen bond stretching frequency of a complex in solution. *Nature* **257**, 41–43 (1975).
5. Shelby, R. M., Harris, C. B. & Cornelius, P. A. The origin of vibrational dephasing of polyatomic molecules in condensed phases. *J. Chem. Phys.* **70**, 34 (1979).
6. Steiner, T. The hydrogen bond in the solid state. *Angew. Chem. Int. Ed.* **41**, 48–76 (2002).
